# Direct imaging and electronic structure modulation of moiré superlattices at the 2D/3D interface

**DOI:** 10.1038/s41467-021-21363-5

**Published:** 2021-02-26

**Authors:** Kate Reidy, Georgios Varnavides, Joachim Dahl Thomsen, Abinash Kumar, Thang Pham, Arthur M. Blackburn, Polina Anikeeva, Prineha Narang, James M. LeBeau, Frances M. Ross

**Affiliations:** 1 Department of Materials Science and Engineering, Massachusetts Institute of Technology (MIT), Cambridge, MA USA; 2 Research Laboratory of Electronics, Massachusetts Institute of Technology (MIT), Cambridge, MA USA; 3 John A. Paulson School of Engineering and Applied Sciences, Harvard University, Cambridge, MA USA; 4 McGovern Institute for Brain Research, Massachusetts Institute of Technology, Cambridge, MA 02139 USA; 5 Department of Physics and Astronomy, University of Victoria, Victoria, BC Canada; 6 Department of Brain and Cognitive Sciences, Massachusetts Institute of Technology, Cambridge, MA 02139 USA

**Keywords:** Surfaces, interfaces and thin films, Electronic properties and materials, Two-dimensional materials, Imaging techniques

## Abstract

The atomic structure at the interface between two-dimensional (2D) and three-dimensional (3D) materials influences properties such as contact resistance, photo-response, and high-frequency electrical performance. Moiré engineering is yet to be utilized for tailoring this 2D/3D interface, despite its success in enabling correlated physics at 2D/2D interfaces. Using epitaxially aligned MoS_2_/Au{111} as a model system, we demonstrate the use of advanced scanning transmission electron microscopy (STEM) combined with a geometric convolution technique in imaging the crystallographic 32 Å moiré pattern at the 2D/3D interface. This moiré period is often hidden in conventional electron microscopy, where the Au structure is seen in projection. We show, via ab initio electronic structure calculations, that charge density is modulated according to the moiré period, illustrating the potential for (opto-)electronic moiré engineering at the 2D/3D interface. Our work presents a general pathway to directly image periodic modulation at interfaces using this combination of emerging microscopy techniques.

## Abstract (web summary)

Here, advanced scanning transmission electron microscopy techniques are used to image the atomic structure at the interface between 2D MoS_2_ and 3D Au nanoislands, revealing a moiré superlattice and illustrating the potential for (opto-)electronic moiré engineering at the 2D/3D interface.

## Introduction

Following the success of moiré engineering in modulating (opto-)electronic properties of graphene/hexagonal boron nitride (hBN) heterostructures^1,2^ and twisted bilayer graphene^3–6^, studies have extended the moiré toolbox to include systems such as double bilayer graphene^7^, trilayer graphene^8^, and van der Waals (vdW) heterostructures composed of transition metal dichalcogenides^9,10^ and hBN-graphene-hBN stacks^11,12^. Recently, moiré engineering has been extended beyond vdW heterostructures, to 3D/3D oxides^13^. Moiré engineering is yet to be utilized for tailoring the quasi-vdW interface between a 2D material and 3D metal. Engineering such 2D/3D interfaces is key to device applications where 2D materials make contact, through a well-controlled junction, to a 3D material such as a metal or semiconductor^14–16^. In contrast to 2D/2D heterostructures, moiré engineering at the 2D/3D interface requires consideration of the stacking of atomic planes in the out-of-plane direction. 3D stacking introduces an additional tuning parameter in 2D/3D systems for modulating moiré properties that is not available in 2D/2D heterostructures^17^.

The ability to image moiré superlattices directly is required to map electronic property modulation onto atomically-resolved structure^18^. Various techniques have been used to observe moiré superlattices. These include reciprocal space imaging via low energy electron diffraction^19,20^ and convergent beam electron diffraction (CBED)^21^; spatially resolved property measurement via scanning tunneling microscopy (STM)^22,23^, atomic force microscopy (AFM) modalities^2,24^, near-field optical microscopy^13^, and infrared nano-imaging^5^; and imaging of transmitted intensity via high-resolution and dark field (scanning) transmission electron microscopy, (S)TEM^25,26^. Of these techniques, STM and (S)TEM are the only two that exhibit real-space atomic resolution. STM is widely used to characterize moiré patterns in 2D materials on bulk substrates, such as graphene on Ru^27^, Ir^28^, and Cu^29^. However, STM measurements are challenging for deeply buried interfaces and for the suspended layers that are gaining traction in 2D device physics^30,31^. (S)TEM, on the other hand, provides detailed information for suspended moiré systems fabricated from solely 2D materials^25,26^. Interpretation is more challenging for 2D/3D interfaces due to the necessity of considering the 3D structure of layers away from the interface^32,33^. This has resulted in discrepancies in periodicity measurement between imaging techniques^22,33^. The MoS_2_/Au{111} system highlights these challenges, with different values reported for the periodicities of superlattices measured via STM and (S)TEM, 32 Å and 18 Å respectively^22,33^.

To reconcile such discrepancies and map moiré structure-property relations at the 2D/3D interface, we combine an analytic convolution technique and a range of STEM imaging techniques, integrated differential phase contrast (iDPC) and four-dimensional (4D) STEM, to decouple the spectrum of higher order moiré patterns. We investigate MoS_2_ /Au{111} as a model 2D/3D system, relevant to TMDC (opto-) electronics^14^, and also examine hBN/Au{111}, relevant in plasmonics^34,35^. iDPC STEM measures the phase of the sample transmission function, enabling direct interpretation as the projected electrostatic potential in thin samples^36–38^. 4D STEM is a rapidly developing technique in which a pixelated array detector is used to collect a CBED pattern at each probe position in the STEM image. The resulting 4D dataset can be filtered post-acquisition to produce reconstructions such as bright field, annular bright field, annular dark field (ADF), ptychography, and iDPC^39^. 4D STEM has been applied to analysis of materials including Cu^40^, ZrO_2_^41^, LiNiO_2_^42^, DyScO_3_^43^, graphene^44^, MoS_2_^45^ and WS_2_^46^, with 2D materials particularly well-suited due to their small thickness^47^. We show that iDPC and 4D STEM are able to decouple higher order moiré periods to form real space images of the moiré pattern at the 2D/3D interface of MoS_2_/Au{111}, revealing the crystallographic 32 Å period. We explain the difference compared to conventional (S)TEM in terms of projection effects of the ABC stacking of the 3D metal. We then use ab initio electronic structure calculations to corroborate that MoS_2_/Au{111} charge density modulation is concentrated at the interface and follows the 32 Å moiré periodicity. Together these findings demonstrate the utility of direct imaging via iDPC and 4D STEM for understanding the structure and electronic properties of 2D/3D heterostructures.

## Results

### Microscopy of the MoS_2_/Au{111} system

**Fig. 1:**
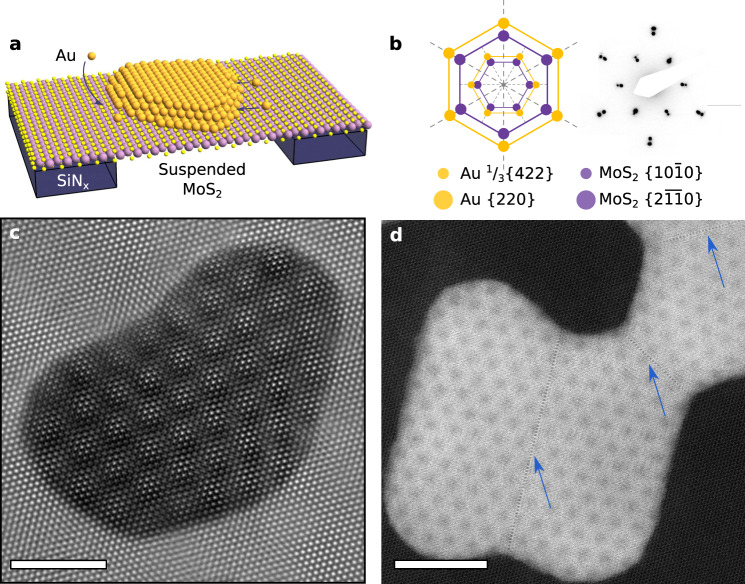
HRTEM and STEM demonstrating epitaxial MoS_2_/Au{111} moiré. **a** Schematic of epitaxially aligned Au deposited on suspended MoS_2_ supported on a SiN_x_ TEM grid. Orange atoms represent Au, yellow S, and purple Mo. The SiN_x_ membrane is shown in dark blue. **b** Reciprocal space model and experimental selected area electron diffraction pattern of the Au {111} zone aligned on MoS_2_ {0001}, with weak intensity ^1^/_3_{422}_Au_ spots (see text) aligned with $\{10\overline 10\}_{\rm{MoS}_{2}}$ and higher intensity {220}_Au_ spots aligned with $\{2\overline {11}0\}_{\rm{MoS}_{2}}$. Orange dots represent frequencies from Au crystal planes, while purple represent frequencies from MoS_2_ crystal planes. **c** HRTEM image of Au nanoisland on MoS_2_, showing apparent 18 Å-period moiré pattern. Scale bar, 40 Å. **d** High angle annular dark field (HAADF) STEM image. Scale bar, 80 Å. Coalescence boundaries are marked by blue arrows.

An example of the MoS_2_/Au{111} interface is shown in Fig. 1. In contrast to the mechanical transfer processes employed for fabricating vdW heterostructures, the 2D/3D systems studied here were formed by direct epitaxial growth^48^ in ultra-high vacuum conditions (Methods). The resulting samples consist of flat, faceted Au{111} nanoislands with an average edge length of 25 nm and height of 8 nm (Fig. 1a, Supplementary Fig. 1) that are epitaxially aligned on suspended MoS_2_{0001} (Fig. 1b), with uniform moiré periodicities across micrometre-scale areas. Selected-area electron diffraction (SAED) confirms 0° rotation between Au and MoS_2_ with a standard deviation of 0.2° (Supplementary Fig. 2). In Fig. 1b and other SAEDs, we observe spots indexed as ^1^/_3_{422} Au reflections. These are classically forbidden for the FCC structure but their presence is consistent with Au nanoisland literature^49^ (Supplementary Note 1). High resolution (HR) TEM shows that the islands are single crystalline, with no evidence of misfit dislocations and grain boundaries (Fig. 1c). The discontinuity in the moiré pattern visible at some boundaries arises from island coalescence. Here, both rigid body displacements and twin boundaries arise from stacking faults between coalesced islands (Fig. 1d, blue arrows).

The uniform moiré periodicity and sinusoidal intensity modulation show that the Au and MoS_2_ lattices are undistorted in the plane of the interface, even near island edges. This is different from the case of twisted vdW structures, which frequently display reconstructions^25,26^. The absence of distortion can likely be attributed to weak quasi-vdW bonding at the MoS_2_/Au{111} interface^14^. Motion of islands at room temperature is consistent in suggesting loose binding of the Au nanoislands to the underlying substrate (Supplementary Movie 1). During their motion, the islands exhibit rotation up to 0.3°, visually amplified in the angle of the moiré pattern (Supplementary Fig. 2). These MoS_2_/Au{111} interface characteristics are consistent for a range of Au thicknesses and uniform across samples (Supplementary Fig. 3).

### Moiré site inequivalence

**Fig. 2:**
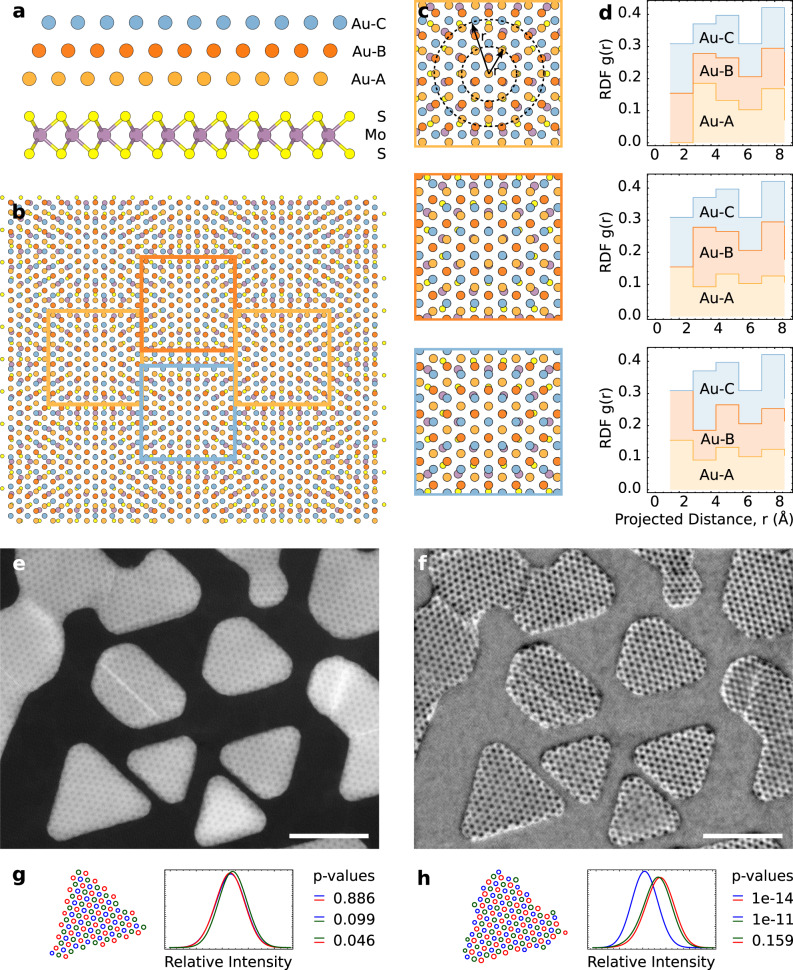
Atomic models, RDFs, HAADF, and iDPC characterization of 32 Å moiré structure. **a** Atomic model [100] zone axis for the 32 Å moiré with the Au atoms indicated (orange, red, blue) to highlight relative stacking of A, B, and C sites. **b** Plan view atomic model for the 32 Å moiré. Boxed areas represent three inequivalent sites in the 32 Å moiré. **c** Close up plan-view image of each of the sites highlighted in (**b**). *r* is the projected distance from the central aligned sites, and the dotted circles show two representative *r* values. **d** Corresponding RDFs of the three inequivalent sites in the 32 Å moiré. **e** HAADF and **f** iDPC STEM images showing the (apparent) 18 Å and 32 Å moiré cells, respectively. Scale bars 200 Å. **g**, **h** Relative intensity distributions and statistical variation of the three inequivalent sites in the island immediately above in the corresponding images. The equivalent disk radius for each spot was calculated and partitioned to inequivalent sites (red, green, blue). The histograms were smoothed using a gaussian kernel of radius 0.5 Å for visual clarity.

At first glance, the period of the MoS_2_/Au{111} moiré superlattice in Fig. 1c, d is 18 Å. While this is in agreement with previous HRTEM studies^33^, it is a consequence of the projective nature of conventional (S)TEM imaging. To illustrate this, one can consider a thought-experiment in which the out-of-plane coordinate of the 3D Au{111} structure is ignored; this results in a “projected” hexagonal Au lattice with atomic spacing of 1.66 Å, which indeed yields a moiré pattern of 18 Å with the MoS_2_ substrate. A more accurate view of electron scattering through the Au crystal requires us to include the full face-centred cubic (FCC) Au structure, as shown in Fig. 2a, b. Consider a location where an Au atom from the A layer (orange) is directly above a pair of S atoms, as in the centre of Fig. 2c-top. This site repeats every 32 Å, shown by the orange squares in Fig. 2b. Sites that appear similar (red and blue squares in Fig. 2b) instead have Au atoms from the B or C layers above the S atoms (Fig. 2c-middle, 2c-bottom). The inequivalence of the three sites can be further illustrated via radial distribution functions (RDFs), which show the quantitative difference in atomic locations (Fig. 2d). Although HRTEM (Fig. 1c) and STEM (Figs. 1d, 2e, g) do not distinguish the three sites, we find that iDPC STEM imaging (Fig. 2f), sensitive to the projected electrostatic potential^50,51^, shows small changes in contrast that are statistically significant (Fig. 2h, Methods) and are confirmed by multislice simulations (Supplementary Fig. 4). iDPC can therefore detect the true 32 Å moiré cell at the MoS_2_/Au{111} interface. However, although this modulation is qualitatively and statistically observable, the translation and rotation of the quasi-vdW islands, as in Supplementary Movie 1, preclude a quantitative analysis.

**Fig. 3:**
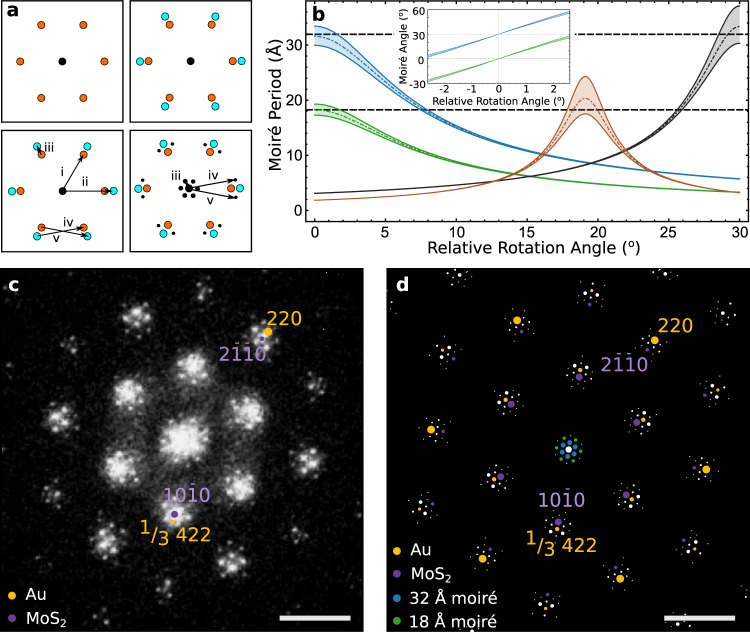
Geometric convolution technique to predict moiré spectrum. **a** Schematic representation of satellite spot generation. Spatial frequencies due to a single lattice shown in the top left panel (orange) are overlaid on those arising from a second lattice on the top right (cyan). The convolution of these two sets of spatial frequencies (i, ii) can be understood as the pairwise vectors connecting spatial frequencies of the two lattices (iii, iv, v - bottom, left). These convolutions generate moiré frequencies (iii, iv, v) shown as black dots in the bottom right panel. **b** Calculated moiré period vs rotation angle for the four largest moiré supercells in the MoS_2_/Au{111} system, illustrated for small (±1%) Au lattice strain. Dot dashed lines represent 0% strain, while the two solid lines on either side represent ±1 strain as a bound. Black dashed lines represent the experimentally observed moiré periods from the FFT, two of which (18 Å and 32 Å) are predicted at 0° relative rotation angle. The moirés are color coded according to the reflections they arise from, with blue arising from the $\{2\overline {11}0\}_{\rm{MoS}_{2}}$: {220}_Au_, green $\{10\bar 10\}_{\rm{MoS}_{2}}$:^1^/_3_{422}Au_,_ orange $\{6\overline {33}0\}_{\rm{MoS}_{2}}$: {642}_Au_, and grey $\{20\overline 20\}_{\rm{MoS}_{2}}$: {220}_Au_ reflections, respectively. The inset shows the variation of moiré angle with relative rotation angle near 0°. **c** FFT of atomic resolution HRTEM image of the MoS_2_/Au{111} image in Fig. 1c showing ^1^/_3_{422} reflection and two visible moiré periodicities around the central spot. Illustrative orange dots represent frequencies from Au crystal planes, while purple represent frequencies from MoS_2_ crystal planes. Scale bar, 0.5 Å^−1^. **d** Simulated FFT for Au/MoS_2_ generated via the geometric convolution technique with each spot colored to show its origin (orange: Au, purple: MoS_2_, blue: 32 Å crystallographic moiré, green: apparent 18 Å moiré). Area of spots is proportional to absolute intensity, but with inner moiré spots magnified 2x for clarity. Scale bar, 0.5 Å^−1^.

Although visible, the iDPC signal from the 32 Å moiré is weak. To consider the full set of spatial frequencies of the Au{111} FCC crystal and obtain a clear real-space image of the 32 Å moiré, we turn to a reciprocal space convolution theorem to predict the entire spectrum of possible moirés in the MoS_2_/Au{111} system (Methods). The geometric interpretation of the convolution theorem indicates that periodicities arise from the pairwise vectors connecting all spatial frequencies of the MoS_2_ and Au lattices^52^ (Fig. 3a). In Supplementary Fig. 5 and Supplementary Table 1, we calculate these periodicities and intensities as a function of rotation angle between the two crystals. The four largest periodicities are shown in Fig. 3b. Additional higher order moirés are also predicted which often exhibit smaller periodicities and weaker intensities (Supplementary Fig. 5). At zero rotation, we indeed recover the 32 Å moiré period, alongside the apparent 18 Å moiré (Fig. 3b). Note that 32 Å moiré periodicity is obscured by the 230% higher intensity reflections of the 18 Å period convolution. We confirm this assignment of moiré periods by showing the experimental fast fourier transform (FFT) of Fig. 1c (Methods, Fig. 3c). The moiré superlattice periods emerge as two sets of satellite peaks around the central beam spot. The simulated diffraction pattern in Fig. 3d is in quantitative agreement with the FFT of the acquired image (Fig. 3c), predicting all the higher order moiré periodicities at the 2D/3D interface.

**Fig. 4:**
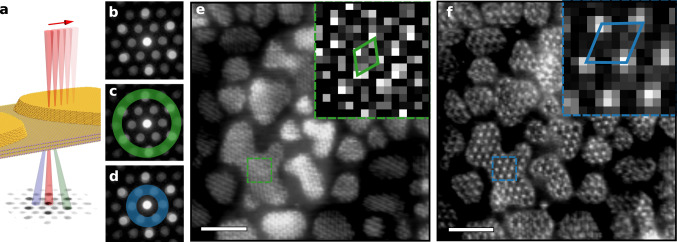
4D STEM imaging of 18 Å and 32 Å moiré periodicities. **a** Schematic of 4D STEM technique showing rastered beam (red) on MoS_2_/Au{111} with corresponding CBED pattern at each point. The green and blue scattered beams are centred on the spots of the annuli shown in (**c** and **d**). **b** CBED pattern formed by averaging patterns collected over the entire scan area, **c** 4D STEM annulus used to isolate 18 Å moiré periodicity (angular range 31–43 mrad, green) and **d** 4D STEM annulus used to isolate 32 Å periodicity (angular range 11–24 mrad, blue). **e**, **f** Virtual ADF STEM images revealing 18 (green) and 32 Å (blue) period moirés, respectively. Scale bar, 200 Å. Insets show unit cells.

To extract a real space image of the weak 32 Å moiré, we employ the technique of 4D STEM (Fig. 4a)^39^. Subsequently we select, with a virtual ADF detector, an annular area of each diffraction pattern to reconstruct an image from the average pattern (Fig. 4b) using certain diffraction spots only. Using an annulus that includes the Au{220} spots and the MoS_2_ {2$\overline {11}$0} spots (Fig. 4c, Methods), we observe the high intensity 18 Å moiré pattern (Fig. 4e). The moiré shows uniform periodicity and sinusoidal intensity modulation across the islands. The symmetry is reduced to periodic line patterns in some areas due to sample tilt, but 18 Å periodicity appears across all islands. If instead we generate a second virtual ADF image using the weaker ^1^/_3_{422} Au and {10$\overline 1$0} MoS_2_ reflections (Fig. 4d), we observe a hexagonal pattern of spots with 32 Å moiré periodicity, consistent with our predictions from geometric convolution and the true crystallographic moiré accounting for 3D structure (Fig. 4f).

### Charge density modulation

**Fig. 5:**
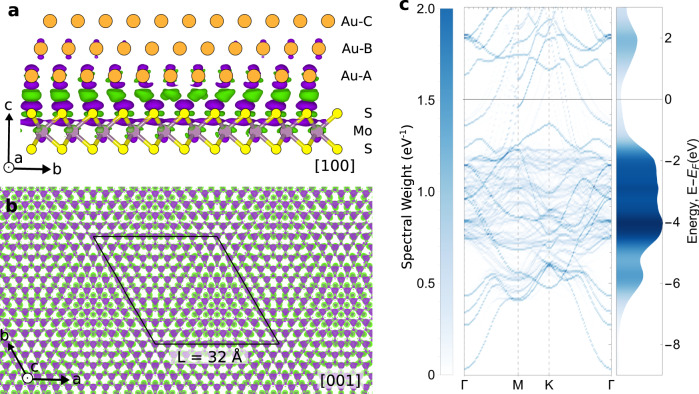
Electronic structure calculations at the 2D/3D interface. **a** Charge density difference viewed down a [100] cross section of the 32 Å commensurate moiré (11_Au_ x 10$_{\rm{MoS}_{2}}$ superstructure). Orange atoms represent Au, yellow S, and purple Mo. Purple denotes negative and green positive charge density isosurface contours. **b** Calculated charge density difference at the MoS_2_/Au{111} interface, as viewed down the [001] axis, showing electronic modulation following the 32 Å moiré periodicity. Black line indicates the 32 Å crystallographic moiré unit cell. **c** Unfolded band structure for MoS_2_/Au{111} system (left) and corresponding density of states (right). Color corresponds to the band’s spectral weight.

To explore the impact of the moiré periodicity on ground state charge density of our 2D/3D structure, we next turn to ab initio electronic structure calculations (Methods). Figure 5a shows a calculated isosurface of ground-state charge density difference for MoS_2_/Au{111} in side-view. The charge density difference is concentrated at the interface, specifically on the upper S layer of atoms, with some penetration to the underlying Mo layer. On the Au side, the charge density difference is concentrated on the first atomic plane, with negligible charge density found in the second Au{111} layer. The charge density modulation due to the 2D/3D interface indeed has a periodicity of 32 Å (Fig. 5b). To quantify the effect of moiré modulation on band structure and density of states, the supercell electronic states can be unfolded onto a single MoS_2_ unit cell (Fig. 5c). Accounting for the 32 Å moiré, band structure calculations are in agreement with prior angle resolved photoemission spectroscopy and scanning tunnelling spectroscopy measurements of the MoS_2_/Au{111} system^53^.

### Application of the method to the hBN/Au{111} interface

To explore the generality of the geometric convolution technique, we also apply it to the hBN/Au{111} structure (Supplementary Fig. 6). Here, the Au lattice is rotated by 10° with respect to the hBN, leading to a more complex situation than the symmetric 0° epitaxy of MoS_2_/Au{111}. This 10° rotation leads to a strong moiré periodicity of 11 Å. Prediction via geometric convolution technique is necessary in these rotated systems to uncover higher order moirés. For this interface, the convolution technique predicts and explains an additional 19 Å periodicity observed experimentally and re-creates the experimental diffraction pattern (Supplementary Fig. 6). The predictions of the convolution technique rely solely on inputs of crystal structure, lattice parameters, and rotation. This analysis, as well as previous literature, illustrate the wide applicability of the technique in moiré analysis^52^.

## Discussion

For the MoS_2_/Au{111} interface, the combination of several different imaging techniques with electronic calculations provides a clearer picture of the moiré structure than is possible with any single measurement. In HRTEM the 18 Å moiré is the strongest visible, leading to the possibility of erroneously predicting that electronic properties should be modulated with this period. Our calculations reveal that electronic modulation instead follows the true crystallographic 32 Å periodicity. This periodicity is hidden in conventional TEM due to projection effects. Instead, for this interface, 4D STEM imaging, combined with a geometric convolution analysis of the full moiré spectrum, allows a direct real-space observational link between atomic structure and moiré-induced electronic modulation at this 2D/3D interface. The combination of analysis techniques also explains the discrepancy between moiré patterns observed by TEM and STM at this 2D/3D interface. These results highlight electronic modulation at the 2D/3D interface, and showcase the growing opportunities for advanced STEM techniques for direct imaging of moiré structures at the atomic scale.

We envision that the coupled application of 4D STEM and the geometric convolution theorem, presented here for analysis of the 2D/3D interface, could also be extended to the direct imaging of higher order moirés in systems with multiple interfaces and could expand opportunities across the field of moiré engineering. Potential applications lie in multiple overlaid moiré superlattices, which have been found to coexist in vdW heterostructures such as hBN-graphene-hBN stacks^11,12^, or in so-called “moiré of moirés” structures arising from relaxation of twisted trilayer graphene and WSe_2_^54^. Although the effects of these coexisting moirés have been reported, they have not yet been directly imaged. This is because the overall moiré observed in HRTEM and STEM is a convolved projection of all the moirés in the system. Using 4D STEM and geometric convolution, moiré characterization could in theory be performed at each interface in the structure by highlighting the relevant diffraction spots. Virtual ADF images could then be used to decouple and directly image each separate moiré. Moreover, 4D STEM could enable simultaneous mapping of crystal orientation, strain, sample thickness, polarization, electric fields, and 3D ptychographic reconstructions of relevant moiré structures^39^. To date, most 2D/3D moiré investigations (including this study) have focussed on epitaxially grown interfaces exhibiting a single orientation. However, future practical development of 2D/3D moiré engineering will require complete control of the structure and orientation of 2D and 3D materials. Emerging fabrication methods using direct transfer of a 3D metal, such as Au, onto 2D materials^55^, or nanomechanical rotation of a 3D nanocrystal using AFM or STM cantilevers^56^, suggest that such control of interfacial orientation is increasingly feasible, extending opportunities of 2D/3D moiré engineering.

## Methods

### Suspended MoS_2_ sample fabrication

Custom TEM chips were fabricated that include a SiN_x_ membrane supported on Si, with 9 holes each 4 µm in diameter. We employed a wedging transfer process to suspend MoS_2_ on these TEM grids^57^. Thermally grown 90 nm SiO_2_/Si wafers were pre-treated with oxygen plasma and MoS_2_ was mechanically exfoliated onto them using the conventional Scotch tape method. Flakes of suitable thickness were identified by their contrast in optical microscopy. A solution of 25 g cellulose acetate butyrate (CAB) in 100 ml ethyl acetate was spin coated onto the sample and baked at 80 °C for 6 min. MoS_2_ flakes were transferred to the TEM grids using a wedging transfer technique^57^. Here, a scalpel is used to cut the CAB around the desired flake. A drop of deionised water can then be intercalated between the CAB and SiO_2_/Si surface and the entire flake transferred to the TEM grid with the CAB polymer handle using a tweezers. The transferred flakes were baked at 140 °C for 5–10 min to improve adhesion. After dissolving the CAB in acetone for 15 min, the flakes were dipped in isopropanol and dried using a critical point dryer.

### Ultra-high vacuum (UHV) epitaxial deposition

To create epitaxial nanoislands, UHV deposition is used. This reduces impurities trapped at the metal-2D interface^58^. The main source of interfacial impurities is polymer residues, which create heterogeneous nucleation sites. Therefore, polymer residue remaining on the 2D material nucleates non-epitaxially aligned nanoislands (Supplementary Fig. 1). The combination of CAB polymer and heat treatment is effective in removing carbon and polymeric contamination^31^; material transferred using other polymers such as PMMA cannot be cleaned as effectively. MoS_2_/SiN_x_ substrates were loaded into a UHV sample preparation chamber and cleaned of residual polymer by heating resistively in UHV to ~550 °C for several hours. Au deposition was carried out in the same multichamber UHV system (base pressure 2 × 10^−9^ Torr), and was deposited in a homebuilt K-cell, using sheet metal placed in a BN crucible, at a rate of 0.5 Å/min. The deposited thickness was calibrated by measuring the evaporation rate with a quartz crystal microbalance immediately before and after deposition. AFM analyses of island thickness were performed in a Veeco Metrology Nanoscope V in tapping mode. There is no intentional heating during deposition, but thermocouple measurements show that the sample temperature rises to 50–60 °C.

### TEM imaging and data analysis

A field-emission TEM (JEOL 2010F) was used for selected area electron diffraction and bright-field imaging, operated at 200 kV. HRTEM imaging was performed with a Hitachi HF-3300V with CEOS BCOR imaging aberration corrector, operated at 60 kV. Figure 1c was obtained from a drift corrected mean of 25 images, where each image was an 8 second exposure, so the total exposure on to the camera was 200 seconds. The electron flux was 500 e^−^/Å^2^/sec so the final image exhibited a total ~100,000 e^−^/Å^2^. Drift tracking over the images gave an average drift of < 7 pm/sec, although most images exhibited less drift. FFTs and line-scans were obtained using Fiji ImageJ software. FFTs of the real-space image are used for observing moiré peaks instead of SAED patterns since moiré peaks were not readily observed at the energies (80–300 keV) used in TEM^59^. The FFT in Fig. 3c was produced by multiplying the source image (Fig. 1c) by a Hanning window prior to taking its FFT to minimise streaking incurred due to the hard edge of the image.

### Multislice image simulations

STEM image simulations in Supplementary Fig. 4 were performed using an orthorhombic supercell consisting of 3 Au layers on an MoS_2_ monolayer (7956 atoms), sliced along the [001] direction. A repeating unit from the supercell was cropped and simulated using custom Python-based STEM image simulation software. Simulation parameters similar to experiments were used, with an accelerating voltage of 60 kV, convergence angle of 24.7 mrad, and collection angles of 25–153 mrad (ADF) and 6–24 (iDPC). Simulated ADF and iDPC images were convolved with a gaussian kernel having FWHM of 80 pm, approximately accounting for the finite effective source size.

### 4D STEM imaging and data analysis

4D STEM imaging was performed with a probe-corrected Thermo Fisher Scientific Themis Z G3 60–300 kV S/TEM operated at 60 kV with a beam current of 50–60 pA in the microprobe mode and a semi-convergence angle of 5.42 mrad, using an Electron Microscopy Pixel Array Detector. The equivalent probe size used in Fig. 4 was ~1 nm and the pixel size was 0.813 nm. Virtual ADF STEM images were generated from the 4D STEM dataset using virtual detectors in the ‘4D STEM Explorer’ program^60^. The HAADF and iDPC images in Fig. 2 were acquired at 200 kV, 25 mrad convergence angle, and a current of 30 pA. The quantification in Fig. 2g,h was performed as follows: input images were convolved with the Laplacian of a gaussian kernel with radius 3.75 Å prior to peak detection; peaks were segmented using a watershed transform and an equivalent disk radius for each spot was calculated and partitioned to inequivalent sites (red, green, blue); a Student *t*-test was used to test the null hypothesis that the different sample means were equal, at the 0.001 significance level.

### Geometric convolution technique

The geometric convolution code was implemented in the computational package Wolfram Mathematica 12.0 and builds on a model previously described for hexagonal lattices^52^. Frequencies arising from the superposition of the two lattice functions were obtained by the convolution theorem, F{t x b} = F{t} ⊗ F{b}, where t and b are the top and bottom lattice functions respectively, F{\,} denotes the Fourier transform, and ⊗ denotes the convolution operation. All possible spatial frequencies arising from observed spots in the SAED/FFT were initially obtained and we make no assumptions in the simulation other than the bulk structure of Au and its {111} orientation. The full set of spatial frequencies of the FCC crystal along the [111] zone axis were used to calculate the moiré periods for all possibilities within the experimentally observed FFT as a function of the relative rotation, while allowing for small (±1%) Au lattice strain. The angles can also be calculated (Supplementary Fig. 2). We then evaluate the most likely candidates to explain the experimentally measured moiré periods and angles (Supplementary Fig. 5).

### Electronic structure calculations

The ground-state charge density difference (Δ*ρ*) between the Au/MoS_2_ heterostructure $(\rho _{{\mathrm{Au}}/{\mathrm{MoS}}_2})$, and pristine Au (*ρ*_Au_) and MoS_2_ $(\rho _{{\mathrm{MoS}}_2})$ is given by

1 $${\mathrm{{\Delta}}}\rho = \rho _{{\mathrm{Au}}/{\mathrm{MoS}}_2} - \rho _{{\mathrm{Au}}} - \rho _{{\mathrm{MoS}}_2}$$

Density functional theory calculations were carried out using the projector augmented wave method implemented in the Vienna ab initio simulation package, VASP^61,62^. We account for the vdW dispersion interactions using the generalized gradient optB86b-vdW functional^63^. We use a cut-off energy of 400 eV on an equivalent Monkhorst-Pack k-points grid of 40x40x1 MoS_2_ unit cell (and similar density supercell). Bandstructure unfolding was performed using the BandUP code^64^.

## Supplementary information

Supplementary Information

Peer Review File

Description of Additional Supplementary Files

Supplementary Movie 1

## Data Availability

The authors declare that the main data supporting the findings of this study are available within the article and its Supplementary Information files.

## Code availability

Code available upon request from the authors.
