# Supplementary material for: Direct imaging and electronic structure modulation of moiré superlattices at the 2D/3D interface
Source: Nat Commun. 2021 Feb 26;12:1290. doi: 10.1038/s41467-021-21363-5 (PMC7910301; doi:10.1038/s41467-021-21363-5)
Supplement: Supplementary file 1 — Supplementary Information [file 41467_2021_21363_MOESM1_ESM.pdf]

Supplementary Information: Direct Imaging and Electronic Structure Modulation of Moiré Superlattices at the 2D/3D Interface

Authors

Kate Reidy[§1], Georgios Varnavides[§1,2,3], Joachim Dahl Thomsen[1], Abinash Kumar[1], Thang Pham[1], Arthur M. Blackburn[4], Polina Anikeeva[1,2], Prineha Narang[3], James M. LeBeau[1], Frances M. Ross[1*]

[1]Department of Materials Science and Engineering, Massachusetts Institute of Technology (MIT), Cambridge, MA, USA

[2]Research Laboratory of Electronics, Massachusetts Institute of Technology (MIT), Cambridge, MA, USA

[3]John A. Paulson School of Engineering and Applied Sciences, Harvard University, Cambridge, MA, USA

[4]Department of Physics and Astronomy, University of Victoria, Victoria, BC, Canada

[§]These authors contributed equally: Kate Reidy and Georgios Varnavides

*Corresponding author: fmross@mit.edu

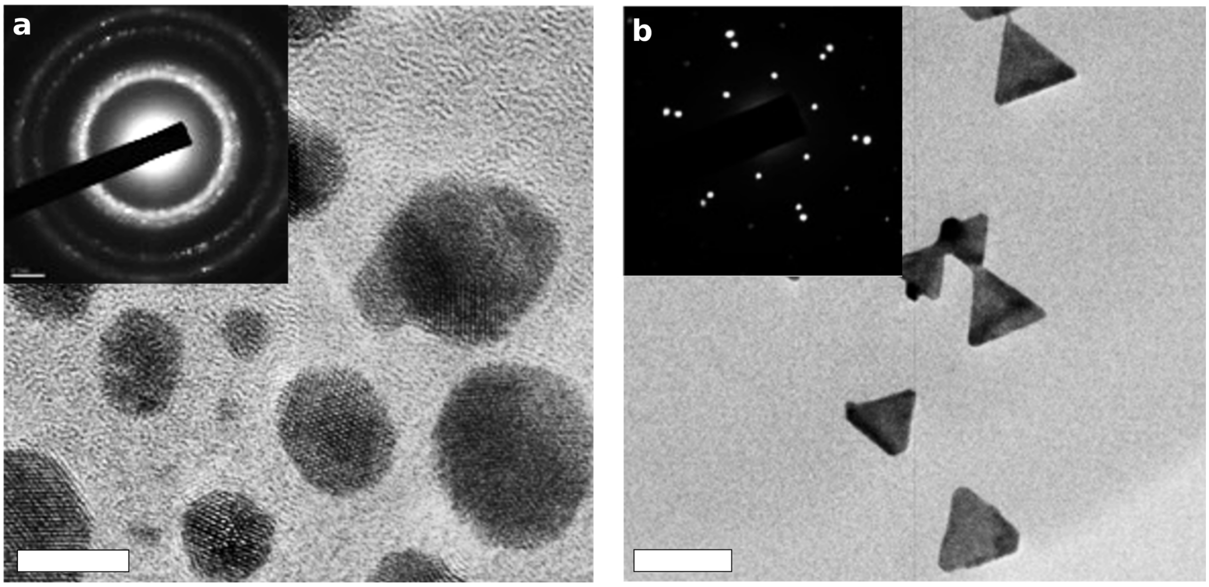

Supplementary Figure 1. Achieving epitaxial nucleation and growth of Au on 2D materials, shown here for graphene. a, Au nanoisland morphologies after deposition onto a 2D material substrate that had not been annealed. Inset shows a polycrystalline diffraction pattern. Scale bar, 50 Å. **b,** Au nanoisland morphologies after deposition onto a substrate that had been annealed in UHV at ~550 ºC for several hours. Scale bar, 500 Å. The images are consistent with polymer residues as the main source of interfacial impurities and hence heterogeneous nucleation sites[1,2,3]. The removal of residue by the pre-anneal results in larger, faceted islands with a 15 x lower nucleation density.

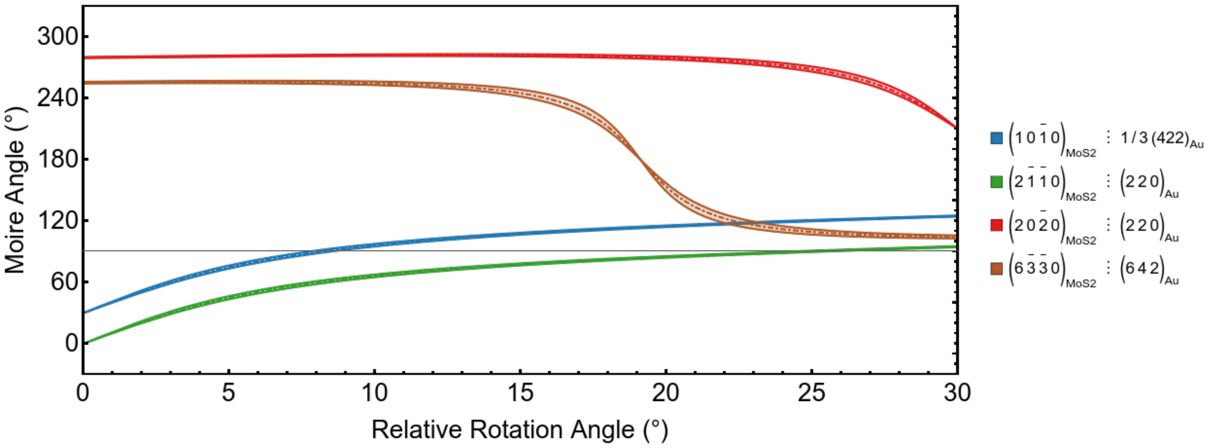

Supplementary Figure 2. Moiré angle vs. rotation angle of MoS_2/Au{111} system. Moiré angles magnify even small misalignments in the underlying lattice[4]. For the 18 Å moiré shown in green, moiré angles of 15° for each 1° rotation between the MoS_2 and Au lattices are possible [5]. Therefore, a ~3° rotation of the moiré pattern in an island corresponds to only a ~0.2° rotation of the island. Most islands are within this 0 ± 0.2° standard deviation window.

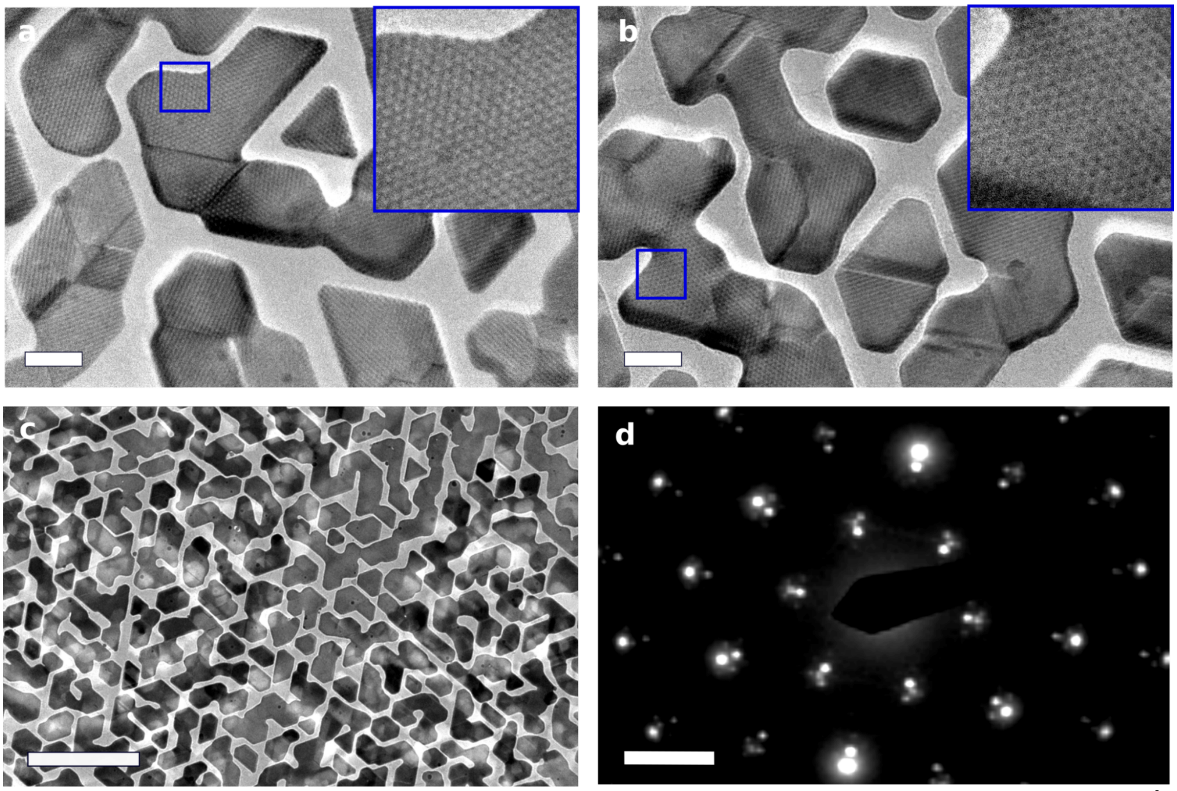

Supplementary Figure 3. Thicker Au{111} deposition. a-c, TEM images of 100-120 Å Au on MoS_2, showing coalesced islands and 18 Å moiré pattern. White shadows are caused by multiple diffracted beams passing through the large objective aperture used. Scale bars 200 Å in a, b, 2000 Å in c. **d,** Diffraction pattern showing Au{111} and MoS_2{0001} spots epitaxially aligned. Scale bar 0.5 $Å^{-1}$.

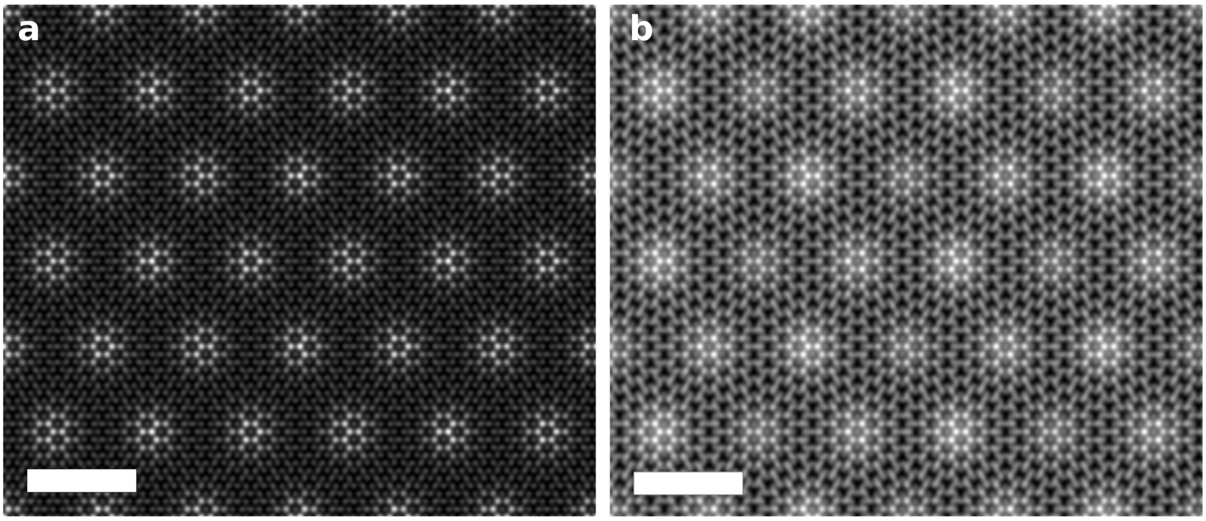

Supplementary Figure 4. Simulated multislice a, ADF and **b,** iDPC STEM images with 3 layers of Au and one layer of MoS_2. The simulated ADF image shows the apparent 18 Å moiré as in conventional TEM, however the iDPC image illustrates a subtle 32 Å moiré. Scale bars 20 Å.

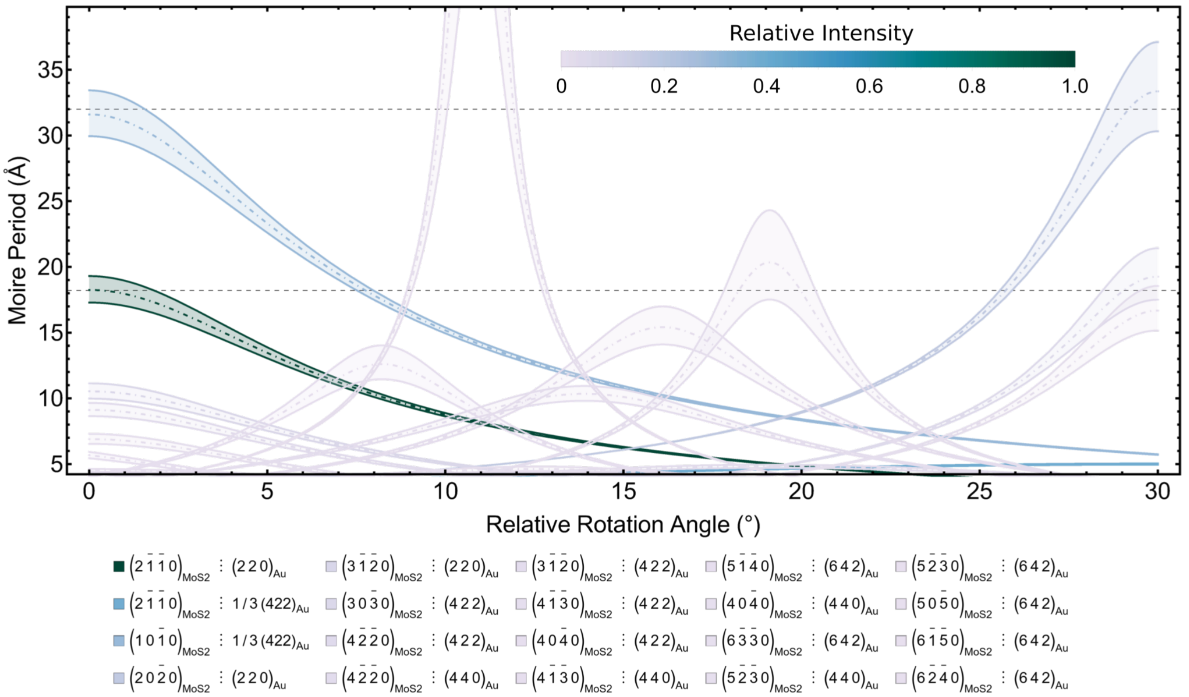

Supplementary Figure 5. Moiré period vs. rotation angle for the spectrum of possible moiré periods in the MoS_2/Au{111} system. An example of the geometric convolution output for the MoS_2/Au{111} system, showing 20 moirés that have a period larger than 4 Å. Of the 147 originally calculated lines, many of the moiré periodicities are very small across the entire spectrum of rotation angle, and only a handful are within the bounds of the experimentally observed moiré periodicity. The moiré bands are coloured according to their relative intensity, further illustrating why the 18 Å moiré is observed in conventional (S)TEM.

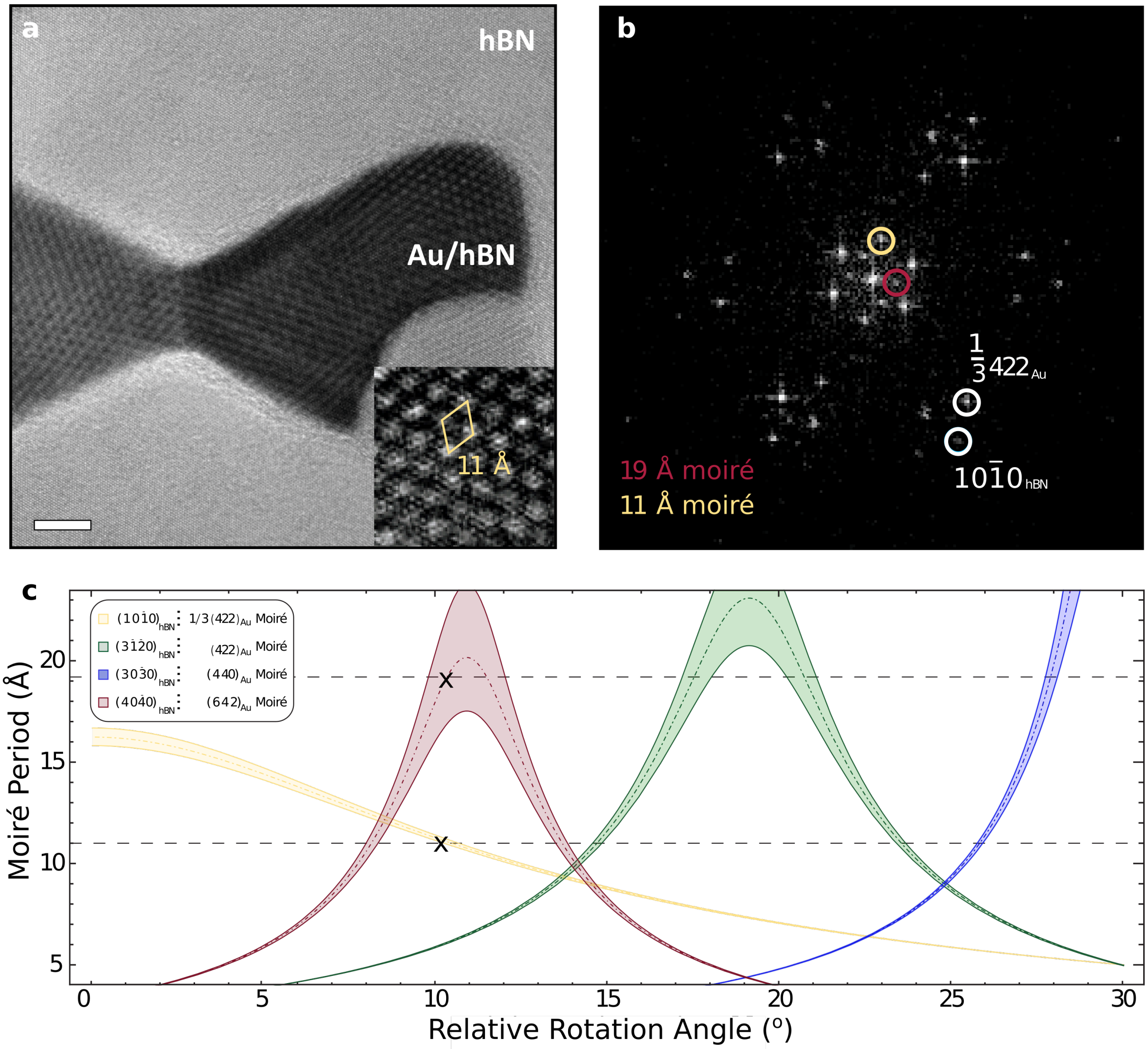

Supplementary Figure 6. Application of geometric convolution technique to hBN/Au{111} system. a, HR-TEM image of two {111} triangular Au nanoislands on hBN, each showing 11 Å period moiré pattern. Scale bar, 50 Å. Inset shows magnified region of 11 Å moiré periodicity. **b,** FFT of the image, indicating the $\frac{1}{3}\{422\}$ forbidden reflection spots and moiré satellite spots. **c,** Subset of possible moiré supercells for Au on hBN vs. rotation angle, determined via the geometric convolution technique. Black dotted lines represent the experimentally observed (11 Å) and FFT-suggested (19 Å) moiré periods. The 11 Å moiré period can be explained by a 10° rotation between Au and hBN. Further, at this rotation we predict another weaker 19 Å period moiré pattern, consistent with the FFT. Geometric convolution analysis determines that, in this case, the forbidden moiré is the dominantly observed moiré, and the hidden moiré occurs as a result of the $\{40\bar{4}0\}$ hBN spots with the {642} spots of Au.

Supplementary Table 1. Relative intensity of moiré patterns for the spectrum of possible moiré periods in the MoS_2/Au{111}system. Moiré intensities as a function of the Au and MoS_2 reflections, showing the 20 highest intensity moirés in MoS_2/Au{111}system and their periods. Intensities for each reflection are assumed to have bulk intensities according to the structure factors of MoS_2 and Au respectively, and we use the convolution theorem to extract the moiré intensities. This gives us access to all but the forbidden $\frac{1}{3}\{422\}_{Au}$ reflections, which we extract by fitting a lorentzian function to a linescan of the experimental diffraction pattern. The 18 Å moiré arising from the $\{2\bar{1}\bar{1}0\}_{MoS2}$: $\{220\}_{Au}$ reflections is the strongest, and normalised to 1. The 32 Å moiré arising from the $\{10\bar{1}0\}_{MoS2}$: $\frac{1}{3}\{422\}_{Au}$ reflections is at 0.3 of this intensity, explaining its lack of visibility in conventional TEM imaging.

MoS_2 Reflection	Au Reflection	Relative Intensity	Moiré Period at φ = 0°. Å
$2\bar{1}\bar{1}0$	220	1	18.2
$2\bar{1}\bar{1}0$	$\frac{1}{3}422$	0.389	3.3
$10\bar{1}0$	$\frac{1}{3}422$	0.298	31.6
$20\bar{2}0$	220	0.172	3.1
$30\bar{3}0$	422	0.062	10.5
$4\bar{2}\bar{2}0$	422	0.040	1.8
$4\bar{2}\bar{2}0$	440	0.028	9.1
$3\bar{1}\bar{2}0$	422	0.022	2.7
$4\bar{1}\bar{3}0$	422	0.009	3.6
$40\bar{4}0$	422	0.006	4.5
$4\bar{1}\bar{3}0$	440	0.006	3.0
$5\bar{1}\bar{4}0$	642	0.005	7.0
$40\bar{4}0$	440	0.004	1.6
$6\bar{3}\bar{3}0$	642	0.003	1.9
$5\bar{2}\bar{3}0$	440	0.003	4.4
$5\bar{2}\bar{3}0$	642	0.002	2.6
$50\bar{5}0$	642	0.001	3.3
$6\bar{2}\bar{4}0$	642	0.0008	5.7
$6\bar{1}\bar{5}0$	642	0.0008	4.0

Supplementary Note 1. Interpretation of $^1/_3\{422\}$ forbidden reflections in Au nanocrystals. The $^1/_3\{422\}$ reflections are 'forbidden' in the sense that they are not visible in conventional structure factor calculations for an FCC lattice. However, experimentally they are often observed in crystals with surface steps, defects, or shape effects and other structure considerations that break the FCC ABC stacking sequence[6–8]. Some models for the presence of these reflections are: 1) Mono-atomic surface steps which cause a non-integer unit cell along the [111] direction, 2) twins in the (111) plane parallel to (111) upper and lower surfaces, 3) twin boundary in the centre of the crystal, 4) high density stacking faults on the (111) plane, and 5) surface reconstructions[9]. In the case of thin platelike Au nanocrystals similar to those grown here, the $^1/_3\{422\}$ forbidden reflections have been shown to be due to shape effects and the projection of higher order Laue zones onto the SAED of the structure[7].

Supplementary References

1. Zan, R., Bangert, U., Ramasse, Q. & Novoselov, K. S. Interaction of Metals with Suspended Graphene Observed by Transmission Electron Microscopy. *J. Phys. Chem. Lett.* **3**, 953–958 (2012).
2. Lin, Y. C. *et al.* Graphene annealing: How clean can it be? *Nano Lett.* **12**, 414–419 (2012).
3. Thomsen, J. D. *et al.* Suppression of intrinsic roughness in encapsulated graphene. *Phys. Rev. B* **96**, 1–8 (2017).
4. Bassett, G. ., Menter, J. . & Pashley, D. . Moiré patterns on electron micrographs, and their application to the study of dislocations in metals. *Proc. R. Soc. London. Ser. A. Math. Phys. Sci.* **246**, 345–368 (1958).
5. Zeller, P. *et al.* What are the possible moiré patterns of graphene on hexagonally packed surfaces? Universal solution for hexagonal coincidence lattices, derived by a geometric construction. *New J. Phys.* **16**, 083028 (2014).
6. Ross, F. M. & Gibson, J. M. Dynamic Observations of Interface Propagation during Silicon Oxidation. *Phys. Rev. Lett* **68**, 1782–1786 (1992).
7. Reyes-Gasga, J., Gómez-Rodríguez, A., Gao, X. & José-Yacamán, M. On the interpretation of the forbidden spots observed in the electron diffraction patterns of flat Au triangular nanoparticles. *Ultramicroscopy* **108**, 929–936 (2008).
8. Bell, D. C. *et al.* Imaging and analysis of nanowires. *Microsc. Res. Tech.* **64**, 373–389 (2004).
9. Germain, V., Li, J., Ingert, D., Wang, Z. L. & Pileni, M. P. Stacking Faults in Formation of Silver Nanodisks. *J. Phys. Chem. B* **107**, 8717–8720 (2003).
