# Supplementary material for: Direct imaging and electronic structure modulation of moiré superlattices at the 2D/3D interface
Source: Nat Commun. 2021 Feb 26;12:1290. doi: 10.1038/s41467-021-21363-5 (PMC7910301; doi:10.1038/s41467-021-21363-5)
Supplement: Supplementary file 3 — Description of Additional Supplementary Files [file 41467_2021_21363_MOESM3_ESM.pdf]

Description of Additional Supplementary Files

Title: Supplementary Movie 1.

Description: Translation and rotation of quasi-van der Waals bonded Au islands on MoS_2. Au nanoislands on MoS_2 recorded in STEM ADF conditions at room temperature. Small translations and rotations of the islands show the weakness of the bond between Au and MoS_2. The beam current was 9.44 pA and the scan area was 115 nm x 115 nm. 1k x 1k images were recorded at 6.31 seconds/frame, 32.5 mrad convergence angle, and 1.125 Å pixel spacing.
