# Supplementary material for: Direct imaging and electronic structure modulation of moiré superlattices at the 2D/3D interface
Source: Nat Commun. 2021 Feb 26;12:1290. doi: 10.1038/s41467-021-21363-5 (PMC7910301; doi:10.1038/s41467-021-21363-5)
Supplement: Supplementary file 2 — Peer Review File [file 41467_2021_21363_MOESM2_ESM.pdf]

Editorial Note: This manuscript has been previously reviewed at another journal that is not operating a transparent peer review scheme. This document only contains reviewer comments and rebuttal letters for versions considered at *Nature Communications*.

Reviewers' Comments:

Reviewer #1:
Remarks to the Author:
The authors have clarified my comments and also other referees' comments, and have substantially improved the manuscript, especially to emphasize its importance in providing a general tool for the moire community. Therefore, I recommend its publication in Nature Communcations.

Reviewer #3:
Remarks to the Author:
The authors have fully answered all my requests. I therefore suggest the manuscript for publication as a very interesting and high impact work.
